# Data-driven decision-making for district health management: a cluster-randomised study in 24 districts of Ethiopia

**DOI:** 10.1136/bmjgh-2023-014140

**Published:** 2024-02-29

**Authors:** Bilal Iqbal Avan, Mehret Dubale, Girum Taye, Tanya Marchant, Lars Åke Persson, Joanna Schellenberg

**Affiliations:** 1 Population Health, London School of Hygiene & Tropical Medicine, London, UK; 2 London School of Hygiene & Tropical Medicine, London, UK; 3 Health System and Reproductive Health Research Directorate, Ethiopian Public Health Institute, Addis Ababa, Ethiopia; 4 Disease Control, London School of Hygiene & Tropical Medicine, London, UK

**Keywords:** Health systems, Cluster randomized trial, Maternal health, Child health, Health systems evaluation

## Abstract

**ABSTRACT:**

**Background:**

Use of local data for health system planning and decision-making in maternal, newborn and child health services is limited in low-income and middle-income countries, despite decentralisation and advances in data gathering. An improved culture of data-sharing and collaborative planning is needed. The Data-Informed Platform for Health is a system-strengthening strategy which promotes structured decision-making by district health officials using local data. Here, we describe implementation including process evaluation at district level in Ethiopia, and evaluation through a cluster-randomised trial.

**Methods:**

We supported district health teams in 4-month cycles of data-driven decision-making by: (a) defining problems using a health system framework; (b) reviewing data; (c) considering possible solutions; (d) value-based prioritising; and (e) a consultative process to develop, commit to and follow up on action plans. 12 districts were randomly selected from 24 in the North Shewa zone of Ethiopia between October 2020 and June 2022. The remaining districts formed the trial’s comparison arm. Outcomes included health information system performance and governance of data-driven decision-making. Analysis was conducted using difference-in-differences.

**Results:**

58 4-month cycles were implemented, four or five in each district. Each focused on a health service delivery challenge at district level. Administrators’ practice of, and competence in, data-driven decision-making showed a net increase of 77% (95% CI: 40%, 114%) in the regularity of monthly reviews of service performance, and 48% (95% CI: 9%, 87%) in data-based feedback to health facilities. Statistically significant improvement was also found in administrators’ use of information to appraise services. Qualitative findings also suggested that district health staff reported enhanced data use and collaborative decision-making.

**Conclusions:**

This study generated robust evidence that 20 months’ implementation of the Data-Informed Platform for Health strengthened health management through better data use and appraisal practices, systemised problem analysis to follow up on action points and improved stakeholder engagement.

**Trial registration number:**

NCT05310682.

**WHAT IS ALREADY KNOWN ON THIS TOPIC**

- Decentralised health systems depend on local data and effective decision-making at the district level.
- Although the District Health Information System 2 initiative has transformed data management, evidence is sparse on how to facilitate data-driven decision-making at the district level.

**WHAT THIS STUDY ADDS**

- We developed the Data-Informed Platform for Health, a health system-strengthening intervention, which promotes structured decision-making by health administrators and managers using local data sources, in 12 districts of Ethiopia.
- Using a randomised study design, we found that the Data-Informed Platform for Health strengthened existing decision-making fora at the district level: it improved data management and appraisal practices, systemised problem analysis to follow up on action points and created a culture of positive engagement among stakeholders.

**HOW THIS STUDY MIGHT AFFECT RESEARCH, PRACTICE OR POLICY**

- The Data-Informed Platform for Health could be adapted for other contexts and levels in the health system, enabling managers and administrators to improve service delivery by making the best use of data.

## Introduction

 Health systems in most low-income and middle-income countries (LMICs) have become devolved and decentralised, shifting decision-making from national to district level. Thus, ‘decision space’ at the district level—the lowest administrative unit for primary healthcare—has expanded.^1 2^ District health systems comprise networks of primary healthcare facilities, including hospitals, and are managed by district health management teams (DHMTs). It is the responsibility of autonomous DHMTs to improve healthcare through responsive, local decision-making.

Rapid advancement in health information systems has accompanied the decentralisation. Governments have committed to strengthening health data management—through enhanced information technology (IT) services and focal development initiatives such as District Health Information System 2 (DHIS2)—rendering local data more available.^3^ Nevertheless, despite better data gathering and expanded decision space, the use of local data in decision-making for maternal, newborn and child health services remains limited in LMICs.^4^

Research, long-established, to strengthen health systems has addressed performance improvement,^5^ supported human resources and financial services, and promoted evidence-based practices.^6^ Facility-level service delivery processes have been a priority, with scant attention paid to health system management—the critical back end to effective service delivery. Evidence on health management interventions to identify and address practice-need gaps is limited, yet building managers’ capacity while embedding technology-based solutions can enhance data use and improve DHMTs’ decision-making.

Our earlier research in LMICs found a limited range of well-developed, contextually embedded processes and tools facilitating data use for comprehensive decision-making by district administrators and health managers.^7^ Among current efforts are cycles of data review and planning through externally facilitated workshops, and focused approaches such as human resource management.^8 9^

Data-driven decision-making appraises all sources of evidence to understand health service challenges and develop solutions and follow-up. District-level Health Management Information System data are underused but possess great promise in relation to the six critical health system components: service delivery, medical supplies, workforce, governance, information and finance.^10^ DHIS2 is a transformative electronic data management system for collecting, validating, storing, analysing and communicating data collected routinely in health facilities. However, there are gaps: for example, databases beyond service delivery and functions for using DHIS data for problem-solving. Decentralisation means that local health administrators need more capacity to analyse and use data for decision-making. Yet the culture of data-sharing and collaborative planning among health stakeholders needs to be enhanced.^11 12^

To address capacity and needs, we developed the Data-Informed Platform for Health (DIPH)—a health management systems intervention, which promotes structured decision-making by district health administrators and managers using local data. The DIPH strategy promotes quality decision-making by: (a) defining themes using a health policy and system framework; (b) reviewing data to identify problems; (c) considering alternative options; (d) value-based prioritising; and (e) using a consultative process to develop, commit to and follow up on action plans. The DIPH package included job aids and guidelines, providing tools and knowledge for structured decision-making using available data. The strategy was conceptualised, developed and tested over 10 years: the theory of change is shown in online supplemental annex Ia, which summarises the problem, solution and logic model. A prototype phase, piloted in three health districts in West Bengal, India, reported multisectoral data-sharing and data-based decision-making by district health management.^13^

Committing to data use for decision-making rendered Ethiopia a pioneer among sub-Saharan African countries. The government’s ‘Information Revolution’ initiative was part of the Ethiopian ‘Health Sector Transformation Plan’ (HSTP; 2015–2020) to nurture a culture of information use and strengthen health system governance.^14^ This remained a priority in the HSTP (2021–2025),^15^ resulting in the Woreda Transformation initiative (districts in Ethiopia are ‘Woredas’) to create high-performing district health offices.^16^ In this context, two co-creation workshops with the Ethiopian Ministry of Health (MoH) and leading local public health institutions highlighted challenges faced by district management, especially in data-driven decision-making. The participants were oriented about the nature of the proposed research solution, and they endorsed the DIPH approach. A collaborative strategy for implementing and evaluating DIPH was finalised, and an advisory group was set up, to support the research within the public health system.

This paper describes the implementation of the DIPH interventions at district level in Ethiopia, and its evaluation using a cluster-randomised controlled trial (RCT) to estimate its effect on health information system performance and data-driven decision-making.

## Methods

Our research was embedded in the Ethiopian district health management system.^17^ A cluster-RCT with pre-comparison and post-comparison design was used. Study districts were ‘clusters’. This section details the study context, DIPH implementation and the evaluation of outcomes.

### Study context

DIPH was implemented in all 24 districts of North Shewa zone in Amhara region, Ethiopia (online supplemental annex Ib). This zone was selected in co-creation workshops with the Ethiopian MoH because of its manageable distance from Addis Ababa and because it was felt to reflect the average functioning of the country’s health system. Districts were categorised into 12 pairs based on (a) performance level based on health outcomes, (b) distance from the zonal capital (Debrebirhan), and (c) the presence of Transform PHCU (Primary Healthcare Unit, a non-governmental organisation working locally on data). In each pair, one district was randomly chosen for the intervention group, the other being for the comparison group (online supplemental annex Ic).

The co-creation workshops clarified that the pre-existing Performance Monitoring Team (PMT) meetings were the main platform for monthly performance reviews, priority-setting and decision-making by DHMT staff. However, PMT meetings were held irregularly, attendees lacked the capacity to conduct them and procedural details were unclear. DIPH supported PMTs by: (a) strengthening the forum for regularity and engagement of government and non-governmental stakeholders (health and non-health) to identify challenges, find solutions, and assess resource allocation and responsibilities, with the aim of consensus-building and collective decision-making; and (b) promoting critical review and regular use of diverse local data (DHIS and other) to understand health system progress.

### Implementation (October 2020−June 2022)

Four 4-month cycles of DIPH were conducted in the 12 DIPH-intervention districts over 16 months. The first two cycles were to embed the DIPH strategy within the system by actively addressing any teething problems with the research implementation team, and the subsequent two cycles were fully integrated into the health system. Effectiveness estimation by the research evaluation team followed this. An additional cycle (20 February−20 June 2022) assessed the sustainability of DIPH without the research implementation team supporting on the ground, and information from this fifth cycle will be reported elsewhere.

Before the first cycle, DHMT staff of intervention districts had 3 days of intensive training supported by the DIPH handbook. The research team (BIA, MD and GT) trained five staff members in each district: the District Health Office head, maternal and reproductive health officer, child and nutrition officer, monitoring and evaluation (M&E) officer, and health information technician (HIT) or their delegates.

A field team was recruited and trained, comprising a coordinator, four support supervisors and a data manager. Each support supervisor was responsible for three districts, which they visited monthly to provide technical support, including inducting, orienting and handholding district stakeholders during the implementation of initial DIPH cycles, participating in monthly PMT meetings and monitoring implementation data.

#### Monitoring

Data were collected by DIPH support supervisors for each 4-month cycle. Synthesised findings were presented periodically to district staff to improve the next cycle.

#### Process evaluation

Qualitative semistructured key informant interviews were conducted in March 2022 with members of DHMTs from five DIPH and five comparison districts. The districts were selected based on their previous year’s performance. In each DHMT, the following personnel were interviewed: head of the Health Office, M&E officer, reproductive and maternal health officer, and HIT. 43 interviews were conducted from 2 March to 28 March 2022. Digital recordings of the interviews in Amharic were transcribed verbatim and translated into English. The transcripts were imported into NVivo and coded under themes related to practices of data use and stakeholder collaboration, and the nature and mechanisms of change of those practices. Key themes included dimensions of data use and stakeholder collaboration, differences in practices between DIPH and comparison districts, mechanisms for changes introduced by DIPH, and the effect of contextual factors on the nature and mechanisms of those changes.

### Outcome assessment

Outcomes were assessed through baseline (September 2020) and endline (March 2022) surveys in all 24 districts. Each survey included a review of District Health Office documents and interviews with the DHMT. Before-and-after comparison of health system outcomes (primary) in intervention and comparison districts assessed changes resulting from DIPH in terms of (1) data management practices, especially health information system performance at the district level, including (a) essential infrastructure for data management, (b) data diversity, (c) reporting timelines, (d) data quality assessment mechanisms, and (e) data use; and (2) practices of data-driven decision-making in relation to district-level governance, including (a) evidence-based decision-making, (b) participatory decision-making, (c) understanding data value, (d) health system support for data use or data-driven decision-making, and (e) accountability. The key secondary outcome was the perception of data use culture, a composite index of 33 items encompassing six aspects of decision-making culture: evidence-based decision-making, emphasis on data quality, use of information, problem-solving, responsibility and motivation to use data. For the cluster-randomised trial overall, with 60 participants per arm, equally distributed across 12 clusters, the study had 80% power to detect a 22% increase in the intervention arm compared with the control arm, assuming a 50% proportion of primary outcomes in the control arm and an intraclass correlation of 0.03, with a 95% confidence level.^18^ Survey respondents included all five eligible staff per District Health Office: head of the District Health Office, the HIT, M&E officer, maternal and reproductive health officer, and child health and nutrition officer. The survey instruments were adapted from the Demographic and Health Survey and Performance of Routine Information System Management (PRISM) tools.^19 20^

Summary indices were developed, with conceptual and statistical underpinning based on standard PRISM tools. Indices were calculated for each of the 24 districts at baseline and endline, ranging from 0% to 100%. PMT practices were assessed at two levels: (a) respondent perception of regularity and types of decision-making in PMTs, and (b) content analysis of PMT records to assess the types of issues discussed. We used a difference-in-differences (DiD) approach to evaluate effectiveness by comparing outcomes in districts that were exposed to the intervention with those that were not. In district-level health system research, there is always a potential risk of competing initiatives or programmes existing in the study area, which might introduce bias if a conventional post-intervention comparison is made in an RCT. DiD is a useful analytical technique in such a context to estimate a causal effect with a valid counterfactual. This is because in DiD analysis, the comparability assumption between the two study arms is less strict: in the absence of intervention, differences between study arms will be the same over time. DiD requires assessing change pre-intervention to post-intervention, and comparing this between the two groups. This eliminates biases resulting from trends in the outcome due to causes other than the intervention. All analyses were adjusted for clustering at the district level.

## Results

This section is organised into four sections: (a) establishing comparability of the two study arms, (b) data management practices, (c) practices of data-driven decision-making and (d) perceptions about the culture of data use.

**Figure 1:**
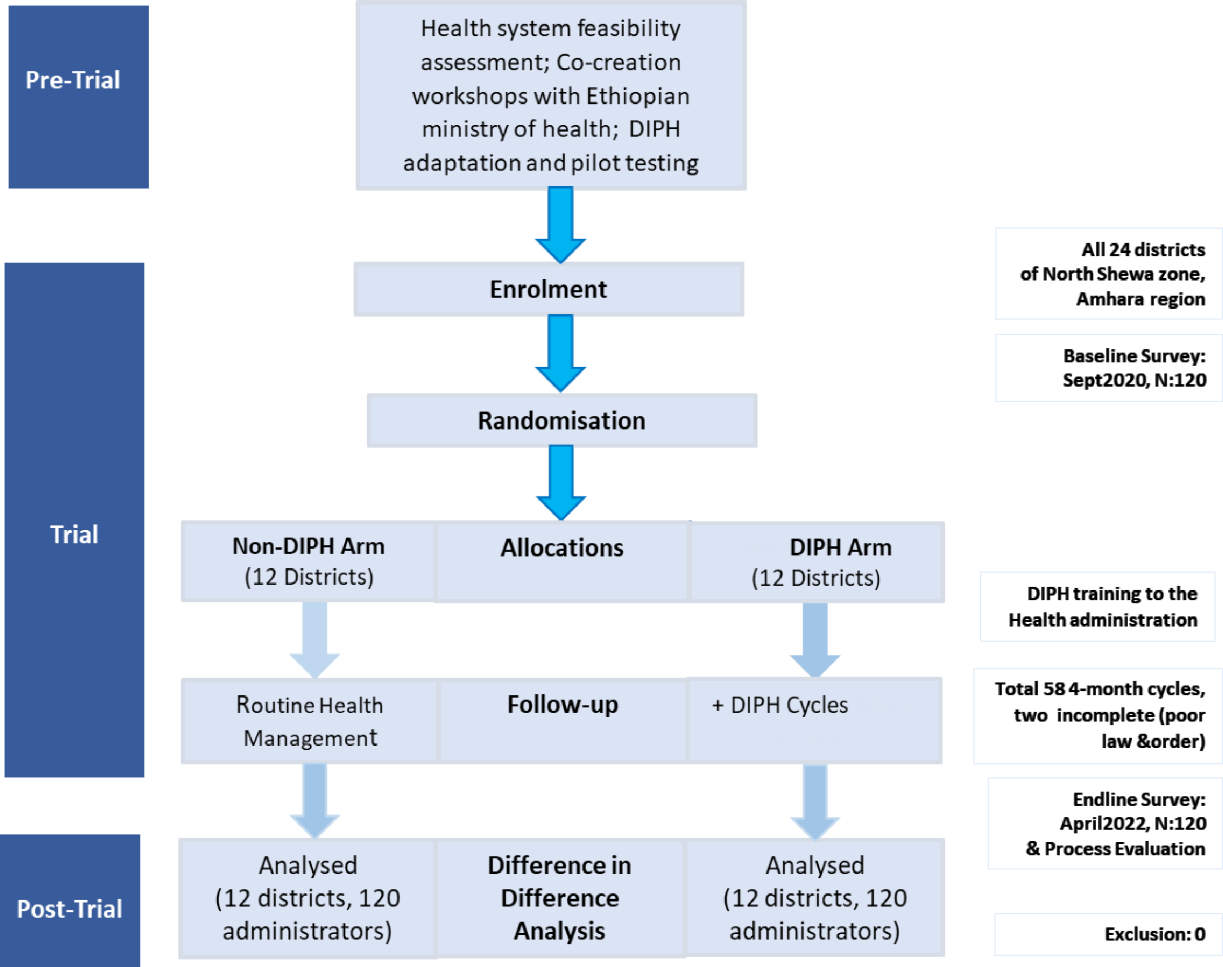
Flow chart of cluster-randomised controlled trial of the DIPH intervention. DIPH, Data-Informed Platform for Health.

58 4-month DIPH cycles were completed across 12 intervention districts between October 2020 and June 2022 (figure 1). In one district, security concerns prevented the initiation of two cycles. The diverse themes included antenatal care, birth attendant skills, community hygiene and various aspects of immunisation (table 1). Over 50% of action points were completed in nearly all (54 of 58, 93%) cycles. The completion rate of the action was at least 80% in most cycles (37 of 58, 63%).

**Table 1:** Total implemented cycles of DIPH in Ethiopia

S no	Theme covered	Cycles
1	Maternal health(ANC 1st, ANC 4th, ANC dropout, syphilis test, PMTCT, iron-folic acid, skilled birth attendant)	31
2	Child health(BCG, penta 1, measles 1, measles 2nd dose, penta dropout, measles dropout, <5 children treated for pneumonia, decrease sepsis infection rate)	16
3	Nutrition(<2 years growth monitoring, <5 years screened for acute malnutrition)	5
4	Hygiene(improved latrine coverage)	2
5	Infectious diseases(HIV testing rate, HIV testing yield, TB referral linkage, malaria incidence)	4
	Total number of completed cycles	58

ANC, antenatal care; DIPH, Data-Informed Platform for Health; PMTCT, prevention of mother-to-child transmission; TB, tuberculosis.

### Comparability of study arms

Sociodemographic characteristics of respondents were summarised by study arm over the study period (table 2). There were 60 respondents, on average, in each of the two arms, five from each district. The average age of respondents was 33 years, the mean professional experience in the health department was 11 years and the mean duration of service in the same district was 6 years. Over time, respondents were relatively more academically qualified, and positive gender changes were observed in both study arms.

**Table 2:** Characteristics of study participants and districts by study arm

Variables	Indicator	Comparison		Intervention	
		Baselinen=60	Endlinen=60	Baselinen=60	Endlinen=60
		%	%	%	%
Respondent designation	District Health Office head	20	20	20	20
	M&E case team head	18	17	18	18
	Health information officer	20	20	20	20
	Others	42	43	42	42
Gender	Female	17	23	13	20
	Male	83	77	87	80
Academic qualification	Diploma	40	32	27	20
	BSc and above	60	68	73	80
		**Mean (SD)**	**Mean (SD)**	**Mean (SD)**	**Mean (SD)**
Professional experience (years)	Respondent age	33 (8)	32 (7)	33 (7)	34 (7)
	Professional experience	12 (11)	11 (7)	10 (7)	11 (7)
	Duration of experience	6 (5)	6 (4)	5 (5)	6 (5)
Characteristics of the study districts	Health centres	4 (1)	4 (1)	4 (1)	4 (1)
	Health posts	17 (7)	18 (7)	17 (6)	19 (7)
	Private for-profit health facilities	5 (4)	6 (5)	3 (2)	7 (5)

M&E, monitoring and evaluation.

The configuration of health facilities in the DIPH and comparison arms was similar. The availability of resources related to data management—including local data storage devices, continuous grid electricity supply, mobile network and access to the national data server—improved comparably in both study arms between baseline and endline, except for wi-fi internet access which improved by 27% in the intervention districts and only 7% in comparison districts.

### Data management practices

Three critical aspects of data management were explored and verified by direct observation: (a) resources (availability of trained staff and MoH guidelines), (b) completeness and record-keeping of key reports, and (c) practices of data quality assessment and data analysis (online supplemental annex IIb).

**Figure 2:**
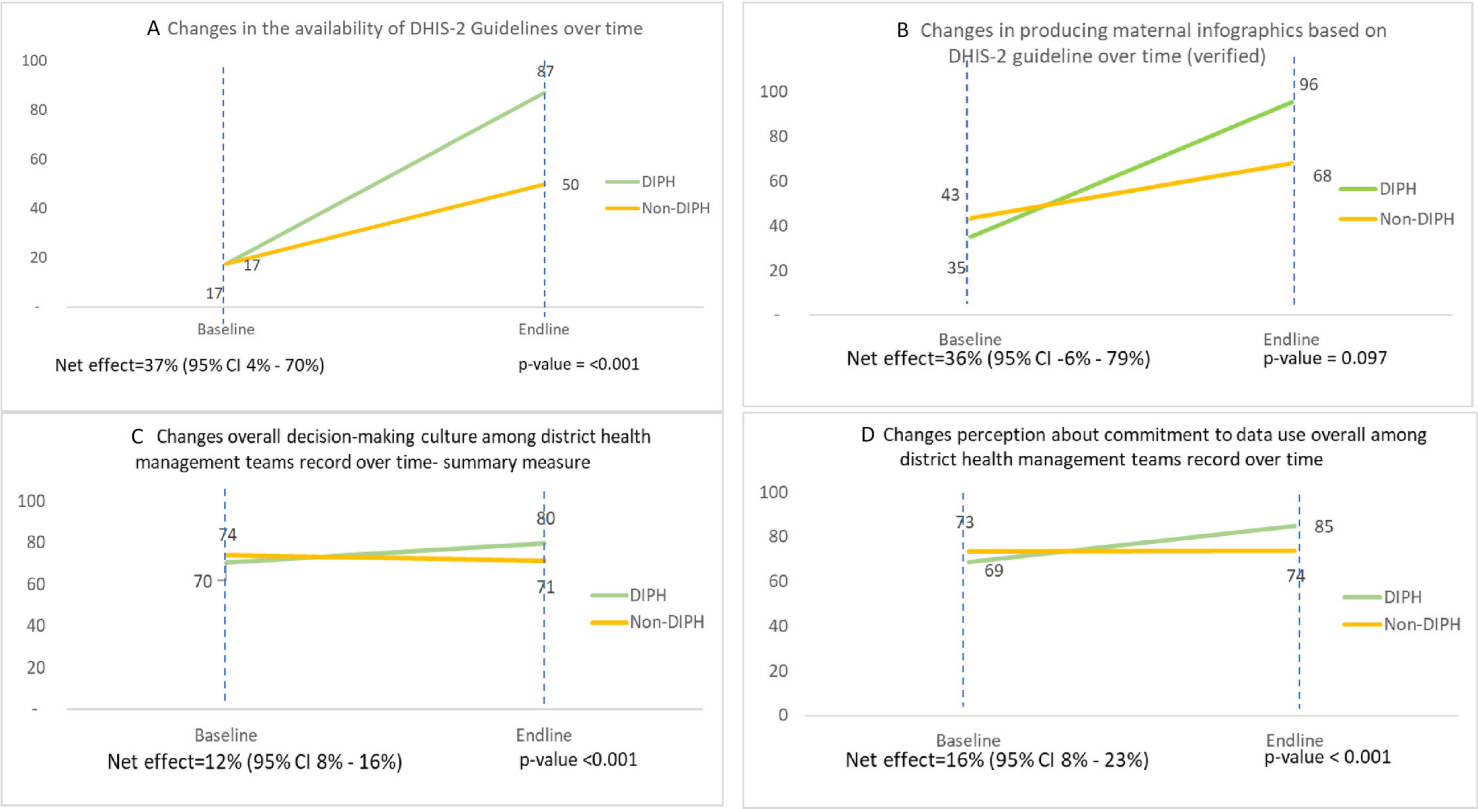
Practices of data management and perception of data use culture. (A) Changes in the availability of DHIS2 guidelines over time. (B) Changes in producing maternal infographics based on DHIS2 guideline over time (verified). (C) Changes in overall decision-making culture among district health management teams’ record over time (summary measure). (D) Changes in perception about commitment to data use overall among district health management teams’ record over time. DHIS2, District Health Information System 2; DIPH, Data-Informed Platform for Health.

Availability of written guidelines or manuals for data review and data quality was considerably improved in DIPH districts: for example, the availability of DHIS2-specific data management guidelines rose from 17% (baseline) to 87% (endline) in intervention districts, as against 17–50% in comparison districts, resulting in 37 percentage points more in the intervention than in comparison arms (95% CI: 4%, 70%) (figure 2A).

Data processing and analysis practices improved in both arms, but changes were greater in the intervention districts. The practice of producing up-to-date maternal infographics from DHIS2 was observed in less than half of the districts at baseline. There was a 36 percentage point greater improvement in intervention districts than in comparison districts (figure 2B).

In the process evaluation, it was reported that the digital interconnectedness of information systems including interoperability introduced by DIPH—especially optimal linkages to DHIS2—resulted in the functional advantage of district management teams taking an integrated view of service delivery challenges and decision-making processes.

### Practices of data-driven decision-making

**Figure 3:**
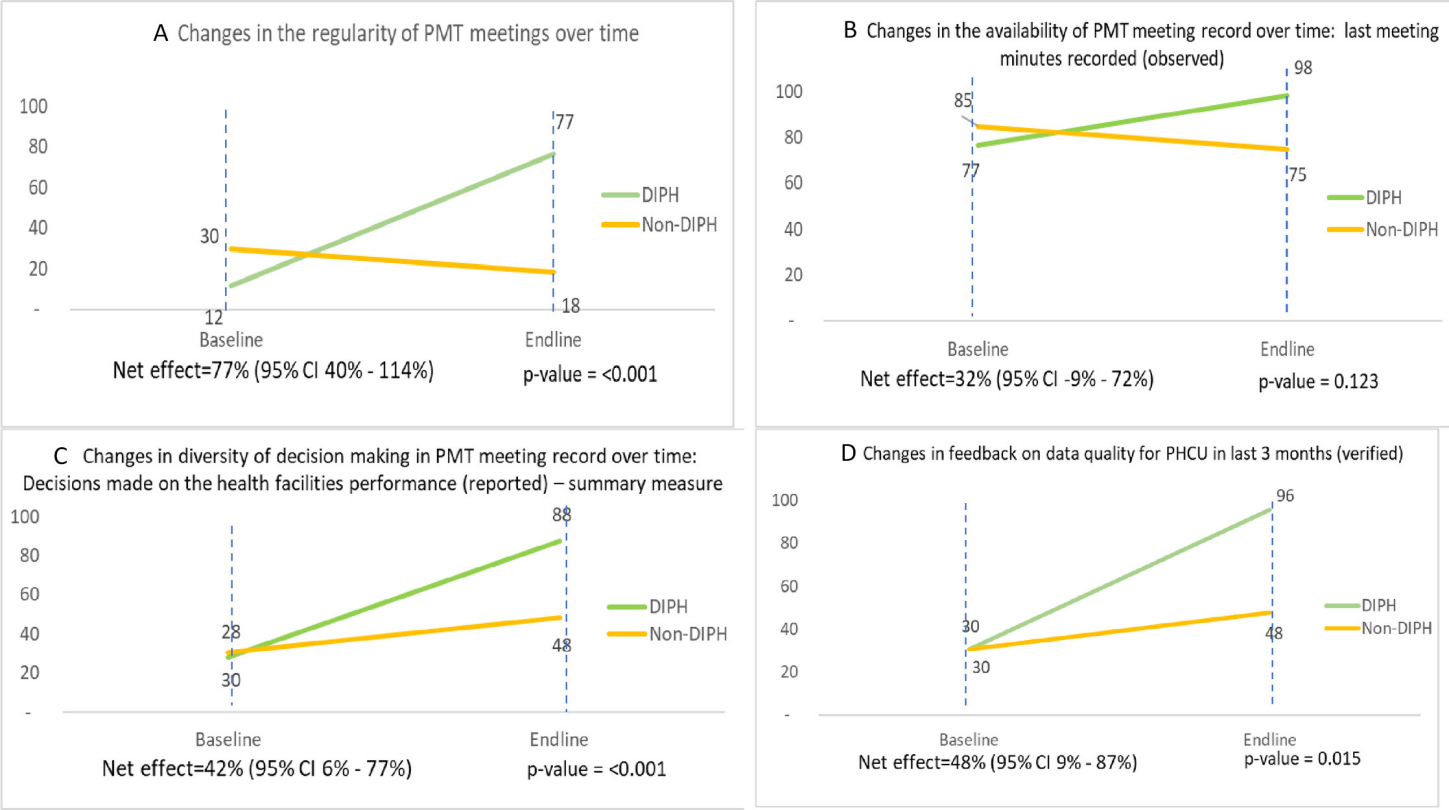
Practices of data-driven decision-making. (A) Changes in the regularity of PMT meetings over time. (B) Changes in the availability of PMT meeting record over time: last meeting minutes recorded (observed). (C) Changes in diversity of decision-making in PMT meeting record over time: decisions made on the health facilities’ performance (reported)—summary measure. (D) Changes in feedback on data quality for PHCU in last 3 months (verified). DIPH, Data-Informed Platform for Health; PHCU, Primary Healthcare Unit; PMT, Performance Monitoring Team.

In the intervention districts, holding regular PMT meetings in the last 3 months increased from 12% to 77% between baseline and endline, with a fall from 30% to 18% in comparison districts, resulting in DiD of 77 percentage points (95% CI: 40%, 114%) (figure 3A). Keeping of minutes in the latest PMT meeting increased from 77% at baseline to 98% at endline in intervention districts, yet fell from 85% to 75% in the comparison districts (figure 3B).

In the process evaluation, all intervention districts reported an increased regularity of PMT meetings. The adoption of DIPH procedures for data use and planning, use of the PMT logbook and strengthened ownership of the process also enhanced commitment to the meetings.

DIPH helps us to easily access information and deliver quality data by introducing the software. It is helping us to timely conduct review meetings by better coordination among district management members. (Head of DIPH District Health Office)

In the last recorded PMT meeting minutes, the types of decision-making were noted and a summary measure was developed to assess the diversity of decision-making: themes included coverage of specific services, human resource management and emerging issues (online supplemental annex IIa–c). DiD analysis showed a 42 percentage point greater increase (95% CI: 6%, 77%) in the intervention districts over time, suggesting diverse decision-making with regard to assessing performance and problem-solving (figure 3C). At the end of the study period, providing feedback on data quality to primary healthcare facilities (PHCU) based on PMT proceedings was almost universal in intervention districts as against only 48% in comparison districts, with a resulting DiD of 48 percentage point positive change (95% CI: 9%, 87%) in the intervention group (figure 3D).

### Perceptions of data use culture

The decision-making culture improved over time in intervention districts from 70% to 80%, with little change in the comparison districts: the DiD estimate suggested a net 12 percentage point improvement as a result of the intervention (95% CI: 8%, 16%) (figure 2C). Decision-making culture reflects the values and beliefs of district health administrators regarding collection, analysis and use of information to improve performance. Among its domains, the largest gains were in the perception of commitment to data use, with a net 16% (95% CI: 8%, 23%) improvement over 18 months (figure 2D). However, no meaningful effect was observed in acknowledging and rewarding good performance within district management teams (for further details, see online supplemental annex IId).

In the process evaluation, respondents reported changes in their abilities in data use and collaborative decision-making culture as a result of DIPH activities:

DIPH training empowered us to closely monitor down to the lowest level of the healthcare system, and in a fixed time frame. Responsibilities are shared among all stakeholders. We continually assess whether activities have met their target and set action points for those that didn’t. Now, everyone does their job with a sense of accountability which was lacking before. (M&E officer, DIPH District Health Office)

Data use and collaborative procedures introduced by DIPH have been adopted in other district-level review meetings and routine tasks. It has especially influenced managers, who consult data before making decisions. Analysis comes before making a decision, and I believe this is one of the results of the DIPH training. (IT officer, DIPH District Health Office)

## Discussion

We promoted structured decision-making by district health administrators and managers through their use of local data by embedding a new approach in their routine work. The DIPH intervention improved competencies in reviewing data, identifying problems by health system blocks, prioritising issues and implementing practical solutions through integrated, collaborative decision-making. The most remarkable results were observed in monthly PMT meetings. Each meeting, without exception, discussed the quality of data reported by the health facilities, resulting in structured feedback to the facilities on their performance, and formulated theme-specific action points which were followed through to completion. In addition, statistically significant effects were observed in the beliefs and opinions of district health administrators and managers: for example, their commitment to improving local data quality and promoting evidence-based decision-making.

The key DIPH mechanisms of change (online supplemental annex Ia) in data use and stakeholder collaboration were observable during implementation. These included training, supervision and a tools package, leading to improvements in data quality and use in PMT meetings—both in the participation and contributions of stakeholders, and in collaborative decision-making. Data quality improved through group reviews, regular screening for errors in DHIS2 and reporting. Data use improved by strengthening analysis skills, digital use and data-sharing, and through the PMT meetings themselves. Stakeholders’ contributions were enhanced through formalised roles and task assignments, joint planning, follow-up, the digital platform and enhanced data analysis, as well as planning and implementation skills. Collaborative decision-making improved in terms of consensual decisions and task-sharing. The DIPH intervention worked in synergy with DHIS2 in the effective use of data for performance review and problem-solving at the local level: to the best of our knowledge, no similar tool or strategy exists in the DHIS2 platform.

Our study took place in a context of health system decentralisation in Ethiopia. Institutional and national governance contexts affect the embedding and sustainability of any health system management intervention. In the Ethiopian devolved health system, the national MoH still issues policy directives. Nonetheless, districts have sizeable space for evidence-based decision-making to interpret and operationalise national directives. The relevance of approaches such as DIPH is commensurate with the degree of autonomy available to district health teams. The role of district management is crucial in translating national and regional policies into action while addressing local health needs, including resource management, staff supervision, coordination among projects and services, and responsiveness to community needs and performance targets. Each aspect requires evidence-based decision-making and commitment to follow-up.^21^ Among the obstacles to reaching population health targets in the primary healthcare system are limited core competencies and a lack of system-level opportunities to enable quality decision-making by district managers.^22^

DHMTs work within broader sociocultural, organisational, structural and relational norms.^23^ In addition to structural changes—that is, decentralisation and devolution—behavioural hierarchies and power dynamics set the tone for interactions, collaborations and decision-making boundaries among stakeholders. DHMTs possess a unique understanding of their local health context: with improved compilation and interpretation of local data, and tools for collaborative decision-making, DHMTs can devise the most practical and feasible solutions for their local healthcare system. Collaboration among district stakeholders for joint decision-making was a key theme of the DIPH intervention. Diverse members of the district cabinet—including the district administrator, deputy administrator and sectoral heads—were contacted for participation.

DIPH is fundamentally an application of ‘systems thinking’ principles in that data-driven decision-making is explicitly and critically linked with subcomponents of the health system. District managers reviewed available routine data with consideration to organisational and population contexts, and found feasible solutions within their means, bringing efficiency to their district’s primary healthcare performance.^24 25^ Unlike linear thinking, which assumes a single cause-and-effect model, systems thinking breaks down the complexity of a problem into manageable, interconnected components, incorporating multiperspective, participatory and iterative approaches while defining solutions.^26^ Despite its promise, there is negligible evidence of systems thinking being translated into health system processes and district-level decision-making.^27^,^29^

While use of technology can enhance the efficiency of DIPH implementation, it is not a prerequisite for its success. As exemplified by our initial paper-based prototype testing in West Bengal, DIPH can be effectively implemented using traditional manual methods.^13^

A proactive, consolidated management tier is central to building an effective and efficient health system.^25^ Most health management initiatives in LMICs, especially in sub-Saharan Africa, focus on core competencies and skills, while lacking evidence on systems operations and contextual dimensions necessary to embed and sustain their interventions.^30^

There have been other recent attempts, contemporary with DIPH, to strengthen district health management, especially in African settings including Ghana Essential Health Interventions Programme, Tanzania Essential Health Interventions Project and African Health Initiative.^31^,^34^ Only a few have undergone rigorous evaluation leading to peer-reviewed publications. Notable among these are PERFORM (2011−2015)^35^ and PERFORM2 scale (2017–2021),^31^ carried out in Ghana, Malawi and Uganda to strengthen the capacity of district health managers—in particular, to develop better human resource management and health system strategies to improve performance. The broader PERFORM strategy was based on an action research cycle delivered in workshop-style formats involving four steps: plan (analyse the primary causes of health service delivery challenges); act (prioritise problems and solutions, design human resource management strategies linked to health systems); observe (follow up on implementation for 3 months); and reflect (on processes and effects). The evaluation of PERFORM was primarily qualitative, reporting a strengthened workforce management and highlighting the potential for workforce performance improvement. Similarly, a workshop-based approach was explored in Nigeria, where district management teams’ capacity to appraise the quality of local data was enhanced through periodic workshops and intense facilitation.^7^ This approach highlighted that cycles of self-assessment of data quality, review and feedback, enabled through regular district-level workshops along with technical support, can enhance the completeness, accuracy and internal consistency of routine health data.

While sharing with these initiatives a focus on district health management and the introduction of structured processes, the DIPH strategy is distinct in that it was designed to be embedded in existing decision-making fora, strengthening those fora to make optimal use of all available key data sources to appraise challenges and implement solutions.

### Limitations

Our outcomes were limited to practices and performances in data-driven decision-making for health system planning, problem-solving and follow-up on an action plan. Though population-level health outcomes remain the ultimate aim, the translation of health management improvement into population-level effects is a long-term process and beyond the scope and timelines of our research. The field implementation team conducted the baseline survey before DIPH intervention training and random allocation of districts to the study arms. An independent data collection team carried out the endline survey assessment, and this team were unaware of the nature of the intervention and the study arm of each district. Thus, although the study was not blinded, and the respondents themselves were inevitably aware of the intervention, we took steps to minimise potential bias. Furthermore, to mitigate any influence of change in the data collection team, our analyses were based on DiD. Although the DIPH intervention was multicomponent, it is not possible to assess the quantitative contribution of individual components nor how they worked together to result in the observed effect. Nevertheless, we have described above possible mechanisms of change through the process evaluation. Among contextual factors, the COVID-19 pandemic and changing law and order situation in some study districts slowed down DIPH implementation. However, of the 60 cycles planned, 58 were completed. Stakeholder participation in PMTs increased over time in both study arms, which may have been due partially to government reforms aiming to promote data use for better district planning and management.

The cost of implementation—that is, financial resources needed across the design, initiation and maintenance stages of DIPH—would have been helpful information for health system stakeholders, especially policymakers, when embedding the strategy.^36^ It is important to note that the intervention is not resource-intensive, as it is about streamlining the decision-making process of the district health administration within an existing routine platform and with existing resources. The key costing element for scaling up would be the training of district administration staff and of supportive supervision, which would be 0.3–0.25 full-time equivalent for each district, particularly during the first months of implementation. The technological aspect would also involve a limited one-off cost. Our lack of costing estimation was a missed opportunity. However, we plan to incorporate a cost estimation in our sustainability research.

Sustainability and scale-up by practitioners and policymakers are of critical importance to health system interventions. In related research, our group has explored broader determinants and frameworks of scalability underpinning the current study.^37 38^ Specifically, additional qualitative research was carried out to assess the possible mechanisms of sustainability and scale-up of DIPH after the assessment reported here. Analysis is in progress and will be shared elsewhere.

## Conclusions

From this robust evaluation of a strategy to enable data-driven decision-making for solving local primary healthcare challenges, we conclude that the intervention improved data management and appraisal practices; systemised problem analysis to follow up on action points; and improved the culture of stakeholder engagement. Further research potential lies in adapting this strategy for various tiers of health systems, with diverse thematic focus.

## Supplementary material

10.1136/bmjgh-2023-014140 online supplemental file 1

## Data Availability

Data are available upon reasonable request.
