# Supplementary material for: Data-driven decision-making for district health management: a cluster-randomised study in 24 districts of Ethiopia
Source: BMJ Glob Health. 2024 Feb 29;9(2):e014140. doi: 10.1136/bmjgh-2023-014140 (PMC10910485; doi:10.1136/bmjgh-2023-014140)
Supplement: online supplemental file 1 [file bmjgh-2023-014140supp001.pdf]

Supplementary file

Annex: Ia

Annex Ia: Figure - Theory of change for the Data-Informed Platform for Health

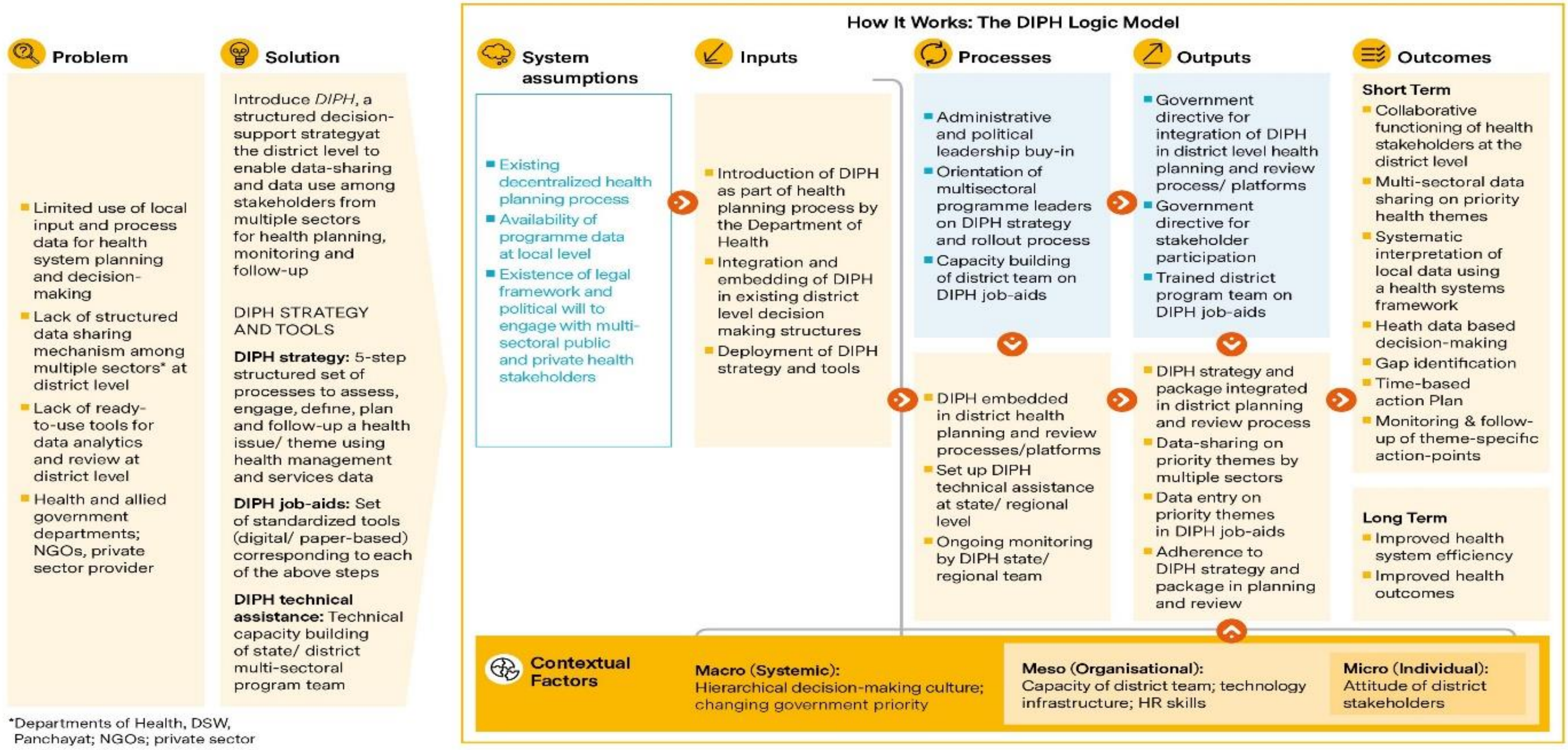

Annex Ib: Map of North Shewa zone, Amhara region of Ethiopia

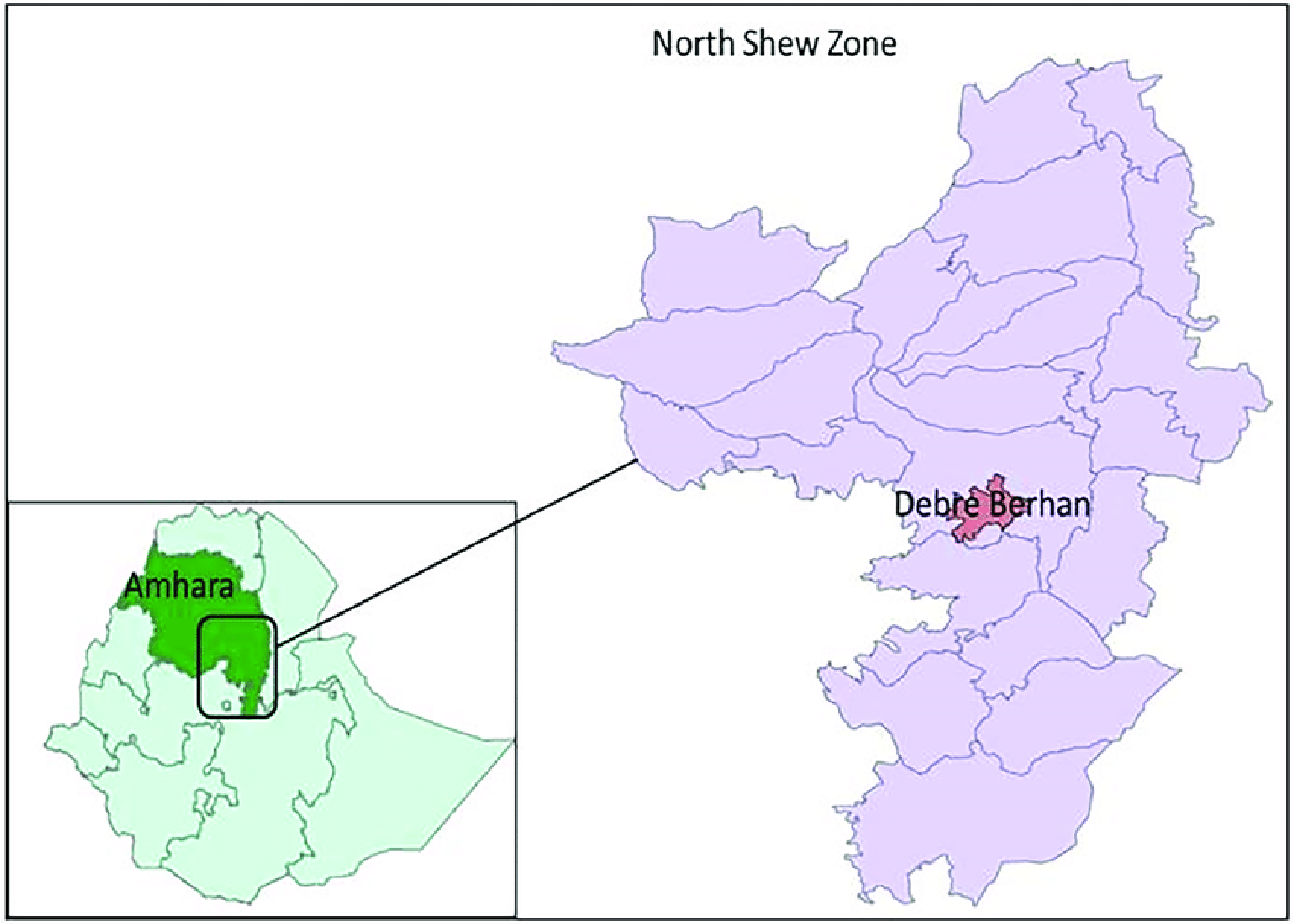

Figure Credit: Deressa T (2018) (CC BY 4.0).

Annex Ic: Table showing criteria and allocations of North Shewa zone districts into intervention and comparison study arms

Intervention Arm (DIPH Districts)					Comparison arm (Non DIPH Districts)				
District Name	Rank	Performance in %	Distance from D/Birhan	Transform Woreda[1]	District Name	Rank	Performance in %	Distance from D/ Birhan	Transform Woreda[1]
Debrebirhan (Urban)	2	64	**0**	**Y**	Shewarobit (Urban)	1	81	**90**	
Bassona worena	16	55	**0**		Ankober	15	55	**42**	
Angolela	9	60	**20**		Siyadebrina Wayu	8	60	**43**	
Tarmaber	11	58	**60**	**Y**	Moretna Jirru	10	60	**65**	
Mojana Wodera	7	60	**72**		Merhabete	4	63	**83**	
Assagirt	20	52	**73**	**Y**	Hageremaryam	22	45	**84**	**Y**
Kewot	3	65	**90**		Menz Mama	2	66	**130**	**Y**
Ensaro	6	61	**133**		Menz Lalo	5	62	**150**	**Y**
Efratana Gdim	12	56	**152**	**Y**	Menz Gera	13	56	**152**	**Y**
Minjarna Shenkora	19	53	**160**	**Y**	Menz Keya	18	53	**184**	**Y**
Berehet	17	54	**180**	**Y**	Midda Woremo	14	56	**183**	
Antsokia Gemza	1	78	**215**	**Y**	Gishe	21	51	**250**	**Y**

[1.] Multisectoral approach by the Ethiopian government to create model districts to achieve SDG goals through priority packaging, partnership promotion and performance tracking.

Annex Id: CONSORT 2010 checklist*: Data Informed Platform for Health (DIPH)

Section/Topic	Item No	Checklist item	Reported on page No**
**Title and abstract**			
	1a	Identification as a randomised trial in the title	1
	1b	Structured summary of trial design, methods, results, and conclusions	3
**Introduction**			
Background	2a	Scientific background and explanation of rationale	4-5
objectives	2b	Specific objectives or hypotheses	5
**Methods**			
	3a	Description of trial design	6
Trial design	3b	Important changes to methods after trial commencement (such as eligibility criteria), with reasons	N/A
Participants	4a	Eligibility criteria for participants	6-7
	4b	Settings and locations where the data were collected	6
Interventions	5	The interventions for each group with sufficient details to allow replication, including how and when they were actually administered	6
Outcomes	6a	Completely defined pre-specified primary and secondary outcome measures, including how and when they were assessed	7
	6b	Any changes to trial outcomes after the trial commenced, with reasons	N/A
Sample size	7a	How sample size was determined	7
	7b	When applicable, explanation of any interim analyses and stopping guidelines	N/A
Randomisation:	8a	Method used to generate the random allocation sequence	N/A
generation	8b	Type of randomisation; details of any restriction (such as blocking and block size)	6, Annex: Ic
Allocation	9	Mechanism used to implement the random allocation sequence (such as sequentially numbered containers), describing any steps taken to conceal the sequence until interventions were assigned	N/A
Implementation	10	Who generated the random allocation sequence, who enrolled participants, and who assigned participants to interventions	6
Blinding	11a	If done, who was blinded after assignment to interventions (for example, participants, care providers, those assessing outcomes) and how	13

	11b	If relevant, description of the similarity of interventions	N/A
Statistic Methods	12a	Statistical methods used to compare groups for primary and secondary outcomes	7
	12b	Methods for additional analyses, such as subgroup analyses and adjusted analyses	N/A
**Results**			
Participant flow	13a	For each group, the numbers of participants who were randomly assigned, received intended treatment, and were analysed for the primary outcome	Figure:1
recommended)	13b	For each group, losses and exclusions after randomisation, together with reasons	Figure:1
Recruitment	14a	Dates defining the periods of recruitment and follow-up	6, Figure:1
	14b	Why the trial ended or was stopped	N/A
Baseline data	15	A table showing baseline demographic and clinical characteristics for each group	Table:2
Numbers analysed	16	For each group, number of participants (denominator) included in each analysis and whether the analysis was by original assigned groups	Annex :II
Outcomes and	17a	For each primary and secondary outcome, results for each group, and the estimated effect size and its	Figure:2-3
estimation		precision (such as 95% confidence interval)	
	17b	For binary outcomes, presentation of both absolute and relative effect sizes is recommended	N/A
Ancillary analyses	18	Results of any other analyses performed, including subgroup analyses and adjusted analyses, distinguishing pre-specified from exploratory	N/A
Harms	19	All important harms or unintended effects in each group (for specific guidance see CONSORT for harms)	N/A
**Discussion**			
Limitations	20	Trial limitations, addressing sources of potential bias, imprecision, and, if relevant, multiplicity of analyses	11-13
Generalisability	21	Generalisability (external validity, applicability) of the trial findings	11-13
Interpretation	22	Interpretation consistent with results, balancing benefits and harms, and considering other relevant evidence	11-13
**Other information**			
Registration	23	Registration number and name of trial registry	3
Protocol	24	Where the full trial protocol can be accessed, if available	Clinical Trials.gov
Funding	25	Sources of funding and other support (such as supply of drugs), role of funders	14

*We strongly recommend reading this statement in conjunction with the CONSORT 2010 Explanation and Elaboration for important clarifications on all the items. If relevant, we also recommend reading CONSORT extensions for cluster randomised trials, non-inferiority and equivalence trials, non-pharmacological treatments, herbal interventions, and pragmatic trials. Additional extensions are forthcoming: for those and for up to date references relevant to this checklist, see www.consort-statement.org.

**Please note that some items have been marked as N/A (not relevant) because the information has been reported elsewhere. The current manuscript is not the main results of the PAL trial (which has been published elsewhere and referred to throughout the manuscript).

Annex Ie: Reflexivity Statement

1. How does this study address local research and policy priorities?

The research, which was implemented in close collaboration with the Ethiopian Ministry of Health, was responding in part to the Government's 'Information Revolution' initiative, as a part of the Ethiopian 'Health Sector Reform Plan' (HSTP 2015-2020 & 2021-2025), to nurture the culture of information use and strengthen district health system governance.

2. How were local researchers involved in study design?

The design and conduct of the study followed a participatory approach involving all of Ethiopia's essential health system stakeholders. Within Ethiopia, the study principal investigator was a co-author from the Ethiopian Public Health Institute, Addis Ababa, who actively collaborated at every stage of the research. With regard to LSHTM staff, a female Ethiopian researcher based in Addis Ababa was responsible for coordinating the research throughout.

3. How has funding been used to support the local research team?

Over 40% of the total project funds were spent in Ethiopia, including salaries for local researchers the data manager and the field team.

4. How are research staff who conducted data collection acknowledged?

The field staff and other stakeholders who collected data and facilitated the project from concept to completion are duly acknowledged in the paper.

5. Do all members of the research partnership have access to study data?

Yes, all members of the research partnership have access to data.

6. How was data used to develop analytical skills within the partnership?

The overall analytical plan was developed collaboratively by the research teams of EPHI (Ethiopia) and LSHTM (UK). Collective team expertise was leveraged for analytical skill development within the project. Weekly meetings were held to familiarise the partners with the statistical methodologies and review analysis of specific aspects of the project data involved in the study.

7. How have research partners collaborated in interpreting study data?

The research team held weekly meetings to discuss data analysis and interpretation, and preliminary findings were presented at several points to get feedback from scientific colleagues and key stakeholders in Ethiopia and within LSHTM.

8. How were research partners supported to develop writing skills?

Senior researchers supported the Ethiopian principal investigator as well as an early-career researcher in refining their scientific writing skills.

9. How will research products be shared to address local needs?

The project was co-created before implementation, and the results were shared in a dissemination workshop with Ethiopian national and regional partners and funders. This paper will be published in an open-access journal, making it accessible to health system researchers in Ethiopia and internationally.

10. How is the leadership, contribution and ownership of this work by LMIC researchers recognised within the authorship?

GT was part of the senior authorship team in developing this manuscript, and MD was instrumental in the data collection and project coordination. LAP was based in Ethiopia throughout the project and gave support and guidance to the implementation team. Separate but related publications from this work, submitted elsewhere, have LMIC team members as lead authors .

11. How have early career researchers across the partnership been included within the authorship team?

Co-authors include early career researcher (MD), and she has contributed to the review and interpretation of the results. Moreover, her doctoral dissertation will include analysis of qualitative data assessing the sustainability of the intervention in Ethiopia, which will also be published in a peer-reviewed journal.

12. How has gender balance been addressed within the authorship?

Three authors are male (BIA, GT and LAP), and three are female (MD, TM and JS).

13. How has the project contributed to training of LMIC researchers?

Two LMIC co-authors were involved at all stages of the research development and execution. They led the study's operational coordination in collaboration with the international investigators. This technical and practical collaboration enabled them to strengthen their research skills.

14. How has the project contributed to improvements in local infrastructure?

The research focus was an intervention which aimed to improve the administrative efficiency of district health systems within their existing data and budgetary resources. Other, more direct financial or material contribution to local infrastructure improvement was beyond the research project's remit.

15. What safeguarding procedures were used to protect local study participants and researchers?

National and international ethics committees approved the research project. It complied with all essential safety and confidentiality procedures. The study did not include any patient or population-level data.

Annex: II

Annex IIa: Table - Availability of general data management resources in the study districts

Variables	Comparison Districts		DIPH Districts	
	Baseline n:12	Endline n:12	Baseline n:12	Endline n:12
	%	%	%	%
Server (FMOH DHIS2 server, if accessible)	58	50	67	58
USB key	83	75	75	83
CD (compact disc)	33	33	25	33
External hard disk	17	17	42	50
Facility mobile phone	75	67	75	83
Internet network	67	58	67	92
Wi-Fi	33	42	67	92
Continuous grid supply	0.0	58	17	50

Annex IIb: Table - Data management status between study arms over time

Variables		Comparison		DIPH	
		Baseline n:12	Endline n:12	Baseline n:12	Endline n:12
		%	%	%	%
**a. Resources for data assessment at the district level**					
Designated person management		100	100	100	100
Designated person received training in the past two years		42	17	33	67
Written guidelines on data entry/compilation (Observed)		50	75	58	92
Written guidelines for Data review and quality control (Observed)		25	58	25	75
**b. Completeness of reporting for last 3 months (by HIT)**					
Keep copies of monthly data reports (CAT)	paper-based copies	100	100	100	100
	electronic copies	0	0	0	0
If Yes, maintain a record of dates of receiving reports from health facilities	Yes	17	92	17	83
**c. Data quality, processing and analysis practices**					
DQA tools use to assess data quality (OBSERVED)		25	42	8	92
In-built electronic data quality validation (OBSERVED)		8	42	8	75
DHIS2 used for Data Entry		100	100	100	100
DHIS2 used for Data Analysis		92	100	92	92
Aggregated/summary DHIS-2 report (OBSERVED)		50	42	58	92
Demographic data on the catchment population(OBSERVED)		67	50	75	83
Comparisons among facilities in the distirct for key DHIS 2 indicators (OBSERVED)		50	50	58	75

Comparisons of annual district/national targets(OBSERVED)	58	33	58.3	75
Comparisons of data over time (OBSERVED)	25	42	33.3	75
Comparisons of service coverage along continuum of care (OBSERVED)	25	50	50	75

Annex IIc: Table - PMT meetings: data-driven decision-making changes over time:

Variables	Comparison		Intervention	
	Baseline (n:60)	Endline (n:60)	Baseline (n:60)	Endline (n:60)
	%	%	%	%
**a. PMT meeting regularity and decision-making**				
Monthly PMT meetings (UNDERSTANDING)	88	92	82	100
Monthly PMT conducted in last three months (PRACTICE)	30	20	12	77
Decisions made on - Formulation of plans	13	48	12	92
Decisions made on - Budget preparation	0	7	3	53
Decisions made on - Budget reallocation	0	5	3	52
Decisions made on - Medicine supply and drug management	10	40	10	87
Decisions made on - Human resource management	8	32	5	98
Decisions made on - Advocacy for policy and programmes	7	33	5	100
Decisions made on - Health services	27	60	37	100
Decisions made on - Promotion of service quality	28	68	22	100
Decisions made on - Reducing the gender gap	3	5	5	35
Decisions made on - Involvement of the community	15	45	12	100
**b. PMT meeting content**				
Performance Monitoring Team conducted (PMT)	100	92	100	100
Minutes of the last PMT recorded (observed)	85	82	78	100
Any discussions on Health Data in the last PMT	40	89	40	100
Any decisions based on the discussions in the last PMT	100	100	100	100
Was action plan formulated in the last PMT (OBSERVED)	25	75	0	92
Any follow-up action taken place on the decisions for the last PMT (OBSERVED)	100	83	0	100
Discussion held in the last PMT- Coverage of service (OBSERVED)	90	100	100	100
Discussion held in the last PMT - Hospital/health center performance (OBSERVED)	70	100	60	92
Discussion held in the last PMT - Disease data (OBSERVED)	20	56	20	83
Discussion held in the last PMT- Identification of emerging issues/epidemics (OBSERVED)	30	89	0	100
Discussion held in the last PMT - Medicine stock-outs (OBSERVED)	20	78	30	83
Discussion held in the last PMT - Human resource management (OBSERVED)	10	44	0	83
Discussion held in the last PMT - Sex-disaggregated data (OBSERVED)	10	11	10	58

Annex IId: Table - decision-making culture

Variables	Comparison Districts				DIPH intervention Districts			
	Baseline (n:60)		Endline(n:60)		Baseline (n:60)		Endline(n:60)	
	Mean	SD	Mean	SD	Mean	SD	Mean	SD
Commitment to quality data	76	14	74	15	74	13	84	9
Commitment to data use	73	14	74	14	69	14	85	8
Practice of evidence-based decision-making	68	8	66	6	64	7	70	8
Practice of problem-solving	72	13	67	13	67	11	78	10
Sharing information between levels	78	13	74	13	76	15	83	9
Sense of duty	78	10	75	11	75	9	80	6
Feeling empowered and accountable	70	15	71	18	66	15	78	10
Rewarding good performance	71	9	74	9	73	9	77	8
Motivation level among staff	76	14	74	15	74	13	84	9

Annex IIe: Table - data-use to create graphs 4 and 5 for the manuscript

	Variables	Comparison		DIPH		Net Effect (95%CI)
		Pre	Post	Pre	Post	
4a	Changes in the availability of DHIS-2 Guidelines over time	17	50	17	87	37% (4% - 70%)
4b	Changes in producing maternal infographics based on DHIS-2 guideline over time (verified)	43	68	35	96	36% (6% - 79%)
5a	Changes in the regularity of PMT meetings over time	30	18	12	77	77% (40% - 114%)
5b	Changes in the availability of PMT meeting record over time: last meeting minutes recorded (observed)	85	75	77	98	32% (9% - 72%)
5c	Changes in diversity of decision making in PMT meeting record over time: Decisions made on the health facilities performance (reported) – summary measure	30	48	28	88	42% (6% - 77%)
5d	Changes in feedback on data quality for PHCU in last 3 months (verified)	30	48	30	96	48% (9% - 87%)
4c	Changes in overall decision-making culture among district health management teams record over time - summary measure	74	71	70	80	12% (8% - 16%)
4d	Changes perception about commitment to data-use overall among district health management teams record over time	73	74	69	85	16% (8% - 23%)
